# Ophthalmic Drug Dosage Forms: Characterisation and Research Methods

**DOI:** 10.1155/2014/861904

**Published:** 2014-03-18

**Authors:** Przemysław Baranowski, Bożena Karolewicz, Maciej Gajda, Janusz Pluta

**Affiliations:** Department of Drug Form Technology, Wroclaw Medical University, Borowska 211A, 50-556 Wroclaw, Poland

## Abstract

This paper describes hitherto developed drug forms for topical ocular administration, that is, eye drops, ointments, *in situ* gels, inserts, multicompartment drug delivery systems, and ophthalmic drug forms with bioadhesive properties. Heretofore, many studies have demonstrated that new and more complex ophthalmic drug forms exhibit advantage over traditional ones and are able to increase the bioavailability of the active substance by, among others, reducing the susceptibility of drug forms to defense mechanisms of the human eye, extending contact time of drug with the cornea, increasing the penetration through the complex anatomical structure of the eye, and providing controlled release of drugs into the eye tissues, which allows reducing the drug application frequency. The rest of the paper describes recommended *in vitro* and *in vivo* studies to be performed for various ophthalmic drugs forms in order to assess whether the form is acceptable from the perspective of desired properties and patient's compliance.

## 1. Introduction

Ophthalmic drug forms have been one of the most important and widely developed areas of pharmaceutical technology for dozens of years. The main reason of continuingly strong interest of scientists in these drug forms is the problem of a low bioavailability of medicinal substance after the application to the eyeball. It is caused by, amongst other reasons, the complicated anatomical structure of the eye, small absorptive surface and low transparency of the cornea, lipophilicity of corneal epithelium, metabolism, enzymolysis, bonding of the drug with proteins contained in tear fluid, and defence mechanisms, that is, tear formation, blinking, and flow of the substance through nasolacrimal duct [1–3]. Low capacity of conjunctival sac, that is, approximately 30 *μ*L without blinking [4], and the aforementioned defence mechanisms cause decrease in drug concentration in the place of application and shorten the time during which the active ingredient stays in the place of absorption. The primary purpose for the development of ophthalmic drug forms is to achieve the required drug concentration in the place of absorption and sustaining it for appropriately long time, which in turn contributes to smaller application frequency [1–5].

One of the first modifications to conventional forms of ophthalmic drugs was introducing polymers to formulation, which enabled longer contact time of active ingredient and the corneal surface, thus increasing its bioavailability. Next possibility to modify the ophthalmic forms active ingredients' bioavailability involved introducing excipients to formulation, which enhanced drugs' penetration into the eyeball. These excipients included chelating agents, surfactants, and cyclodextrins, which, along with active ingredients, form inclusion complexes. This increases solubility, permeability, and bioavailability of poorly soluble drugs [1–4].

The newer drug forms, on which in recent years research has been conducted in order to achieve a controlled release of drug to eyeball tissues, include multicompartment carrier systems, inserts, collagen shields, contact lenses, and the so-called* in situ *gels [1–3, 5]. The advantages of using these new drug forms of controlled release are, among others, increasing bioavailability of substance through extending the time of its contact with cornea—which can be achieved by effective adhesion to the corneal surface, the possibility of targeted therapy preventing the loss of drug to other tissues, ensuring patient's comfort when applying the drug form and during the whole therapy, and increasing resistance to eye defence mechanisms, like tearing [4].

This paper constitutes an overview and characterization of the hitherto developed ophthalmic drug forms.

## 2. Topical Ophthalmic Drug Forms

### 2.1. Liquid Ophthalmic Drug Forms

#### 2.1.1. Eye Drops

Eye drops are accessible in the forms of water and oil solutions, emulsions, or suspensions of one or more active ingredients, which may contain preservatives if stored in multiuse packaging. These forms are sterile and isotonic. The optimum pH for eye drops equals that of tear fluid and is about 7.4. In deciding whether to buffer the drug in this form, one should take into account the stability of active ingredient and the tissue tolerance to the preparation [7–9]. If the pH value gets outside the range of 4–8 which is tolerated by eye, the patient may feel discomfort, there may be irritation, and the drug bioavailability can decrease because of increased tearing [10].

#### 2.1.2. Ophthalmic Solutions

Ophthalmic solutions are sterile, aqueous solutions used for, among other things, cleansing and rinsing eyeballs. They may contain excipients, which, for example, regulate osmotic pressure, the pH, and viscosity of the preparation. They may also contain preservatives if stored in multiuse packaging [7].

#### 2.1.3. Microemulsions

Microemulsions are promising drug forms, inexpensive to produce, and easy to sterilize and stable, providing the possibility to introduce larger amounts of active ingredient.* In vivo* research and clinical examination of healthy volunteers proved extended time periods of effectiveness and increased bioavailability of drugs applied in these forms. The mechanism of action involves the adsorption of nanodrops constituting a reservoir of the drug and the inner phase of microemulsion on the corneal surface, which limits the overflow [5].

Active ingredients for which microemulsions have been developed include difluprednate [11], cyclosporine A [12], flurbiprofen axetil, and the prodrug of flurbiprofen [13].

#### 2.1.4. Modifications of Liquid Ophthalmic Dosage Forms

In the course of technological research on dosage forms, many ways have been proposed as to how to extend the time period of contact of liquid dosage forms with eye tissues, as well as to increase the active ingredient absorption to these tissues. These modifications include the addition of substances which increase viscosity, introducing the drug penetration enhancing substances to formulation, using prodrugs or cyclodextrins [4, 5, 7–10].

#### 2.1.5. Addition of Substances Increasing Viscosity/Adhesion

Extending the time period of contact with cornea and improving bioavailability of substances may be obtained by increasing formulation's viscosity. Substances which have such effect include hydrophilic polymers of high molecular weight which do not diffuse through biological membranes and which form three-dimensional networks in the water. Examples of such polymers include polyvinyl alcohol, poloxamers, hyaluronic acid, carbomers, and polysaccharides, that is, cellulose derivatives, gellan gum, and xanthan gum. The aforementioned carbomer is used in liquid and semisolid formulations as a suspending substance or a substance which increases viscosity, whereas hyaluronic acid is used as a polymer, forming biodegradable and biocompatible matrix, which enables extending time periods of drug release [4, 8].

The research has proved that maximum increase of penetration through the cornea by a solution in the form of eye drops takes place when the viscosity falls into the range of 15 to 150 mPas. An example of “extreme” use of substances increasing viscosity is forming gels, which would enable reducing the frequency of drug application to once daily. It has been proved that synthetic polyoxyethylene-polyoxypropylene block copolymer (poloxamer 407) is suitable for use as a carrier in ophthalmic formulation with pilocarpine, which stimulates the active ingredient. The main disadvantage of this formulation is blurring of vision, which negatively affects its acceptability among patients [4, 8].

Presently, hydrophilic polymers are employed in many ophthalmic products, though rather as compounds that exhibit mucoadhesive properties than for increasing viscosity [4]. These forms contain polymers which connect through noncovalent bonds with conjunctival mucin and usually are macromolecular hydrocolloids with many hydrophilic groups (carboxyl, hydroxyl, amide, and sulfate) able to form electrostatic connections, which enables longer contact with eye surface. Mucoadhesive dosage form is characterized by higher bioavailability in comparison to conventional forms [5]. Examples of polymers which were examined in the direction of mucoadhesion and increasing substance bioavailability in ophthalmic preparations include polyacrylic acid, hyaluronic acid, sodium carboxymethyl cellulose, and chitosan. Other compounds which extend the time period of contact with eye surface are lectins, which were also examined in the direction of selective drug binding to a specified corneal area [4, 5, 8].

Two preparations, NyoGel (Novartis) with timolol maleate and Pilogel (Alcon Laboratories) with pilocarpine hydrochloride, contain cross-linked polyacrylic acids which exhibit mucoadhesive properties, Carbomer and Carbopol, respectively [14].

#### 2.1.6. Addition of Penetration Increasing Substances

The purpose of using penetration increasing substances in ophthalmic drugs is to enhance their corneal absorption by modifying the continuity of corneal epithelium structure. Research has shown that such properties are displayed by chelating agents, preservatives (like benzalkonium chloride), surfactants, and bile acid salts. However, these substances displayed local toxicity, which caused restrictions in their use in ophthalmic drug forms technology [3, 4].

#### 2.1.7. Prodrugs

Modifying drug properties by developing prodrugs also enables increasing drug permeability through the cornea. This method involves modification of chemical structure, which gives the active ingredient new properties, that is, selectivity and site specificity [4]. Examples of medicinal substances for which prodrugs were developed include epinephrine, phenylephrine, timolol, and pilocarpine [4, 15]. Dipivefrine, a diester of pivalic acid and epinephrine, displays seventeenfold higher permeability through the cornea than epinephrine, which is caused by its six hundredfold higher lipophilicity at pH 7.2. Therefore, a smaller dose of dipivefrine applied over the eyeball has similar therapeutic effect to epinephrine. In comparison to conventional eye drops containing 2% epinephrine, eye drops with dipivefrine 0.1% display only slightly smaller activity lowering the intraocular pressure with significant reduction of side effects [15].

#### 2.1.8. Cyclodextrins

Cyclodextrins are cyclic oligosaccharides able to form inclusion complexes with active ingredients, thus increasing the solubility in water of hydrophobic compounds without changing their molecular structure [3, 16]. As carriers, they enable keeping hydrophobic drugs in solution and transport them to biomembranes surface. In the case of ophthalmic drugs, optimal bioavailability of the active ingredient is obtained at the appropriate concentration of cyclodextrins (<15%) in aqueous eye drops solution [4]. The most often used cyclodextrin in developing forms applied over the eyeball is 2-hydroxypropyl-*β*-cyclodextrin, which does not show irritating effects. Eye drops containing drug inclusion complexes, namely, dexamethasone or pilocarpine with 2-hydroxypropyl-*β*-cyclodextrin, are well tolerated and ensure increased bioavailability in comparison to conventional ones [3].

### 2.2. Semisolid Ophthalmic Drug Forms

#### 2.2.1. *In Situ *Gels (or Sol-to-Gel Systems)

*In situ* gels are viscous liquids, showing the ability to undergo sol-to-gel transitions when influenced by external factors, like appropriate pH, temperature, and the presence of electrolytes. This property causes slowing of drug drainage from the eyeball surface and increase of the active ingredient bioavailability. Polymers employed in developing these drug forms include gellan gum, poloxamer, and cellulose acetate phthalate, whereas active ingredients used in the course of research on* in situ* gels include ciprofloxacin hydrochloride, timolol maleate, fluconazole, ganciclovir, and pilocarpine [3–5, 8].

#### 2.2.2. Eye Ointments

Ointments are semisolid dosage forms for external use, usually consisting of solid or semisolid hydrocarbon base of melting or softening point close to human body temperature. After applying the ointment to the eye, it decomposes into small drops, which stay for a longer time period in conjunctival sac, thus increasing drug's bioavailability. Eye ointments have certain disadvantages—although they are well tolerated and safe, they cause, among other things, blurring of vision and sometimes have irritating effects, because of which they are mainly applied night-time [8].

### 2.3. Solid Ophthalmic Drug Forms

#### 2.3.1. Contact Lenses Coated with Drugs

This drug form can absorb on its surface water-soluble substances, released after applying the drug over the eyeball for a longer period of time. The first and most widely used polymer in the production of lenses was the cross-linked poly(2-hydroxyethyl methacrylate) with small amount of ethylene glycol dimethylacrylate [4, 5]. In recent years, research has been conducted on employing silicon-based lenses [17–20]. Interest in contact lenses still grows, which is confirmed by increase in the number of articles on its use published in recent years. Examples of drugs whose pharmaceutical availability from lenses was researched include timolol [17], ciprofloxacin [18], dexamethasone [19], and cyclosporine [20].

#### 2.3.2. Ocular Inserts

Inserts are solid or semisolid dosage forms without disadvantages of traditional ophthalmic drug forms [5, 21]. They are less susceptible to defence mechanisms like outflow through nasolacrimal duct, show the ability to stay in conjunctival sac for a longer period, and are more stable than conventional dosage forms. Their undoubtable advantages over conventional forms are also accurate dosing, the possibility of slow substance release with constant speed, and limiting its systemic absorption. Moreover, using them enables reduction of the drug application frequency, as well as adverse effects and blurring of vision occurrence [8, 21]. Polymeric materials most often employed in developing inserts include, for example, methylcellulose [22] and its derivatives, that is, hydroxypropyl methylcellulose (HPMC) [8, 21–23], ethylcellulose [22, 24, 25], polyvinylpyrrolidone (PVP K-90) [8, 21, 25], polyvinyl alcohol [8, 23], chitosan [21] and its derivatives, like carboxymethyl chitosan [22], gelatin [24, 26], and various mixtures of the aforementioned polymers. Employed polymers indicate the division of inserts into soluble, insoluble, and biodegradable. A well-known insert Ocusert (Alza Corporation), built from copolymer of ethylene and vinyl acetate, is an example of insoluble insert, containing pilocarpine as an active ingredient [5, 8, 27]. The main factors limiting the employment of inserts in the therapy are still patients' unwillingness to abandon traditional dosage forms, the feeling of foreign body in the eye, and sporadic failures in using and introducing inserts, such as unnoticed excretion from the eye [4, 5, 21, 27].

#### 2.3.3. SODI (Soluble Ophthalmic Drug Inserts)

SODI are soluble eye inserts in the form of small oval wafers, produced from acrylamide, *N*-vinylpyrrolidone, and ethyl acrylate. After their application to conjunctival sac, they are moistened by tear fluid, and then they soften and adhere to eyeball surface. This dosage form was originally developed for astronauts to apply it in the state of weightlessness. Drug is released from SODI in a pulsational, uncontrolled manner, and the dosage form ensures its prolonged effect. Active ingredients employed in the course of research on SODI include neomycin, kanamycin, atropine, pilocarpine, dexamethasone, sulfapyridine, and tetracaine [4, 28, 29].

#### 2.3.4. Minidiscs/OTS (Ocular Therapeutic System)

Minidisc is a profiled, convex outside, concave from the side of contact with eye surface, dosage form similar to a contact lens with 4-5 mm diameter. Main copolymers from which minidiscs are developed are *α*-*ω*-bis(4-methacryloxy)-butyl poly(dimethylsiloxane) and poly(hydroxyethyl methacrylate). This dosage form may be either hydrophilic or hydrophobic, which enables extended time period of release of water-soluble and poorly water-soluble drugs. Active ingredients employed in research on minidiscs were, among others, sulfisoxazole and gentamicin sulfate [2, 4, 28, 30].

#### 2.3.5. Artificial Tear Inserts

This dosage form is a long, rod-shaped pellet, containing no preservatives and developed from hydroxypropyl cellulose. It is available on the market under the name Lacrisert and is employed in treatment of the dry eye syndrome. After its introduction to conjunctival sac, the insert absorbs water from conjunctiva and cornea, forming a hydrophilic layer, which stabilizes the tear film and moistens the cornea [2, 5].

#### 2.3.6. Collagen Shield

Collagen shields are developed from porcine sclera, whose collagen displays similarities to the one in human cornea. The shields are stored in dry state and hydrated before they are introduced to the eye. The standard collagen shields, applied by an ophthalmologist, are not individually suited to the patient's eyeball and cause certain discomfort due to interfering with vision. Moreover, they may be accidentally excreted from the eye just after introduction [5].

Collagen shields were tested on animal and human models and may be carriers of antibiotics like gentamicin, anti-inflammatory drugs like dexamethasone or antiviral drugs. In comparison to contact lenses and eye drops, the use of collagen shields enabled obtaining higher drug concentration in the cornea and the aqueous humor [4, 30].

More recent dosage forms built from collagen are the so-called collasomes, small pieces of collagen (1 mm × 2 mm × 0.1 mm) suspended in a 1% methylcellulose vehicle. Collasomes show all advantages of collagen shields without disadvantages of the latter [5, 30].

#### 2.3.7. NODS (New Ophthalmic Delivery System)

NODS is a dosage form patented by Smith and Nephew Pharmaceuticals Ltd, consisting of solidified paper handle and a flag from polyvinyl alcohol, containing the active ingredient, attached to the handle with a soluble membrane. A film containing drug separates from the handle at the point of introduction to conjunctival sac and dissolves in the tear fluid, releasing the active ingredient. This system ensures delivery of specified drug dose to the eyeball and increased bioavailability of active ingredient (even eightfold in the case of pilocarpine) in comparison to conventional eye drops. NODS does not contain preservatives and is sterilized with gamma rays [31–33].

#### 2.3.8. Minitablets

Minitablets are biodegradable, solid drug forms, that, after application to conjunctival sac, transit into gels, which extends the time period of contact between active ingredient and the eyeball surface, which in turn increases the active ingredient's bioavailability [34].

The advantages of minitablets include easy application to conjunctival sac, resistance to defence mechanisms like tearing or outflow through nasolacrimal duct, longer contact with the cornea caused by presence of mucoadhesive polymers, and gradual release of active ingredient from the formulation in the place of application due to the swelling of the outer carrier layers [35, 36].

The development of minitablets applied to the eyeball usually involves using polymers, that is, cellulose derivatives, like hydroxypropyl methylcellulose (HPMC), hydroxyethyl cellulose (HEC), sodium carboxymethyl cellulose, ethyl cellulose [35, 37, 38], acrylates [35], that is, polyacrylic acid and its cross-linked forms, Carbopol or Carbomer [34, 35, 37, 38], chitosan [35], starch, for example, drum-dried waxy maize starch [34, 35], and excipients, that is, mannitol [35, 37, 38], performing the function of solubilizate or sodium stearyl fumarate [35, 38] and magnesium stearate [36, 37] with lubricating properties. Minitablets are developed applying the method of direct compression or indirect method, the latter involving tableting the earlier obtained granules. The advantage of indirect method is the dry granulation stage, which increases flow properties of powders often containing bioadhesive polymers, which enables minitablets production on a larger than laboratory scale [34]. Active ingredients from which minitablets were developed include piroxicam [36], timolol [35, 37], ciprofloxacin [34, 35, 38], gentamicin, and acyclovir [35].

### 2.4. Multicompartment Drug Delivery Systems

#### 2.4.1. Nanoparticles and Microparticles

Polymeric, solid, multicompartment drug delivery systems are promising dosage forms for application to the eyeball. With respect to the size of polymeric microvessels, nanoparticles and microparticles can be distinguished, the former's size being from 10 nm to 1000 nm and the latter's, in case of application to the eyeball, from 1 *μ*m to 5–10 *μ*m [4, 5, 8].

Nanoparticles are polymeric carriers, built from biodegradable, biocompatible, natural, or synthetic polymers with often mucoadhesive properties [39–41]. Ingredients used in its development, for the purpose of application to the eyeball, were poly(alkyl cyanoacrylate), polylactic acid, poly(epsilon-caprolactone), poly(lactic-co-glycolic acid), chitosan, gelatin [40–42], sodium alginate [41, 42], and albumin [40–42]. These forms can be divided into nanospheres, the solid, monolithic spheres built from dense polymer matrix, in which the active ingredient is scattered, and nanocapsules constituting reservoirs, built from polymer membrane surrounding the drug in solid or liquid form [40]. The mechanism of drug absorption from nanospheres or nanocapsules after their application to conjunctival sac involves diffusion or degradation of the polymer [8].

The pointed-out advantages of using nanoparticles as an ophthalmic dosage form include increased corneal penetration and a larger dissolution area, which enables improvement of the active ingredient's bioavailability in comparison to traditional eye drops [40]. On the other hand, the main pointed-out limitation of nanoparticles is their low capacity [8].

Medicinal substances for which nanoparticle delivery systems were developed include sulfacetamide, sparfloxacin, levofloxacin, acyclovir, piroxicam, cyclosporine A, flurbiprofen, and pilocarpine [42].

#### 2.4.2. Liposomes

Liposomes are phospholipid drug carriers usually built of phosphatidylcholine, stearylamine, and various amounts of cholesterol or lecithin and *α*-L-dipalmitoyl-phosphatidylcholine [5, 41, 43, 44]. The pointed-out advantages of these carriers are their biocompatibility, biodegradability, amphiphilic properties, and relative intoxicity [4, 5, 41]. However, it is also emphasized that their stability is smaller in comparison to therapeutic systems based on polymers [5, 8, 41] and that their volume in which drug can be contained is limited [8, 41]. Moreover, their large-scale production is expensive and very difficult technologically [8]. Their employment in ophthalmic drug forms enables improvement of bioavailability of applied substance and its protection from enzymes present on the surface of corneal epithelium [43]. It should be emphasized that effectiveness in delivery of the active ingredient from liposomes depends on many factors, that is, encapsulation efficiency, size and charge of liposomes, stability of liposomes in conjunctival sac, or affinity to corneal surface [41, 43]. Liposomes charged positively, in comparison to ones charged negatively and neutrally, display higher affinity to negatively charged corneal surface and conjunctival mucoglycoproteins, because of which they slow down the elimination of active ingredient from the place of application [41]. In order to increase adhesion of negatively and neutrally charged liposomes to corneal or conjunctival surface, introducing liposome suspensions to mucoadhesive gels or coating them with mucoadhesive polymers has been proposed [4].

Active ingredients for which liposomal ophthalmic drug forms were being developed include acyclovir, pilocarpine, acetazolamide, chloramphenicol [43], and ciprofloxacin [44].

#### 2.4.3. Niosomes and Discosomes

Niosomes are chemically stable, built of nonionic surfactants, two-layered carriers used for both hydrophilic and hydrophobic particles, without the disadvantages of liposomes (chemical instability, oxidative degradation of phospholipids, and expensiveness of natural phospholipids) [2, 5, 28, 41]. Moreover, these biodegradable, biocompatible, and nonimmunogenic carriers extend the time period of contact between drug and cornea, which in turn increases drug's bioavailability [41]. Discosomes are modified forms of niosomes, which also may act as carriers for ophthalmic drugs. Their size varies from 12 to 16 *μ*m. Discosomes differ from niosomes in that the former contain the addition of nonionic surfactants, Solulan C24, a derivative of lanolin, which is a mixture of ethoxylated cholesterol (ether of cholesterol and polyethylene glycol) and ethoxylated fatty alcohols (ether of cetyl alcohol and polyethylene glycol). The size of discosomes is their advantage, because of which they do not enter the general circulation. Moreover, the disc shape ensures better fitting of this form into the conjunctival sac [41]. A substantial research has already been conducted on niosomal drug forms for substances, that is, ganciclovir [42], cyclopentolate, or timolol [41].

#### 2.4.4. Dendrimers

Dendrimers are branched, spherical, monodisperse, three-dimensional polymer structures, of specific size, shape, and molecular mass [45–48]. They may be used as carriers, which enclose the active ingredient inside the polymer structure or create, due to the presence of many functional groups (carboxyl, hydroxyl, and amine), electrostatic or covalence bonds with the surface-bound drug [46–48]. It has been proved that polyamidoamine (PAMAM) dendrimers, used as carriers for ophthalmic drugs, extend the duration of active ingredients' effectiveness and increase their bioavailability [48]. Research on using dendrimers as ophthalmic drug carriers was conducted for model substances: the pupil dilating tropicamide and pupil constricting pilocarpine nitrate. The increased bioavailability of these substances after application to the eyeball may be in this case caused by enclosing the drug inside these structures, which results in slower release of the active ingredient. It is also explained by their bioadhesive properties [47, 48].

### 2.5. Other Ophthalmic Drug Forms and Methods of Application

#### 2.5.1. Filter Paper Strips

These are paper strips covered with pigments (i.e., fluorescein or Bengal Red) and used in diagnostics of corneal, conjunctival, or palpebral damage, as well as in diagnosing the presence of microbiological infections and eyeball infection (for example with* Herpes simplex* virus) [5, 49, 50]. Every strip of the Fluorets preparation, sized approximately 5 × 15 mm, contains 1 mg of sodium fluorescein [49]. The strip is usually wetted with a drop of sterile saline solution [49, 50].

#### 2.5.2. Sprays

Sprays are rarely used ophthalmic dosage forms. Active ingredients for which they were developed include cycloplegics, mydriatics, and their mixtures [5, 51], that is, phenylephrine-tropicamide and phenylephrine-tropicamide-cyclopentolate. Before application to the eye, the distance between dosage device and the eyeball should range from 5 to 10 cm. The advantage of using these forms is the possibility of applying the drug on closed eyelid, and the effectiveness of application is approximately the same as in the case of eye drops containing the same ingredients [51]. Results of research conducted by Martini and his associates proved that miotic effect of pilocarpine hydrochloride applied to the eyeball in the form of spray with the active ingredient concentration at 1 to 4% is close to the effect achieved after applying eye drops of 1% concentration, with the volume of dose applied in spray being 5 *μ*L, which was 6 times lower than one applied in eye drops [52].

#### 2.5.3. Ocular Iontophoresis

It is a noninvasive procedure during which ions are introduced to cells or tissues by use of direct current. When iontophoresis is used in pharmacotherapy, the aforementioned ions are charged drug molecules, with positively charged molecule being introduced to tissue from anode and the negatively charged one from cathode. Iontophoresis enables fast, safe, and painless pharmacotherapy and in most cases also obtains high drug concentration in the desired area [5, 53]. Active ingredients that were employed in the course of research on introducing drug using iontophoresis include gentamicin, dexamethasone, ciprofloxacin, and ketoconazole [53], and it is emphasized that applying antibiotics using this method enhances their bactericidal activity [5, 53].

## 3. Examinations of Ophthalmic Drug Forms Properties

Examinations which have to be performed in order to determine the properties may be divided into performed* in vitro* and* in vivo.* The former determine sterility, the pH, clarity of solutions, visual assessment, size of the particles, tonicity/osmolarity, viscosity, amount of substance, amount of preservative, stability, and* in vitro* release [9, 13, 17, 26, 42, 44, 48, 54, 55]. The latter include the Draize eye test and the* in vivo* release [13, 26, 42, 48, 54, 55]. Other distinguished examinations, performed for chosen drug forms, include analysis of ions and oxygen permeability for contact lenses or determination of encapsulation efficiency for multicompartment drug delivery systems and emulsions [13, 17, 42, 44].

### 3.1. *In Vitro *Examinations

#### 3.1.1. Sterility Examination

The basic requirement for drug forms applied on the eyeball is their sterility. Examination of sterility involves inoculation in aseptic conditions of the sample examined on two microbiological media: thioglycolate medium (*fluid sodium mercaptoacetate *or* sodium thioglycolate*), which is used for growth of aerobic and anaerobic bacteria, and medium with hydrolysate of casein and soy (*soya-bean casein digest media*) used for growth of aerobic bacteria and fungi. A thioglycolate medium with an applied sample is incubated at the temperature of 30–35°C, whereas a medium with hydrolysate of casein and soy with an applied sample is incubated at the temperature of 20–25°C for the time not shorter than 14 days. Two methods are distinguished for inoculation of examined material: direct inoculation and a method involving use of membrane filters [9, 54, 55].

The direct inoculation method, as described in Pharmacopoeia, involves transferring the suitable amount of examined preparation to the medium. If a product has antimicrobial properties, such effect of the substance should be neutralized before the examination. Before their introduction to the medium, the ointments should be diluted with a suitable sterile solvent containing the chosen surface active agent. During incubation, the media with introduced samples should be observed at specified time intervals [9, 55].

The indirect method (membrane filtration method) is used when the character of the product enables it. For water and oil solutions, filters from cellulose nitrate are used in which size of pores does not exceed 0.45 *μ*m. For some products, for example, antibiotics, specifically adjusted filters are employed. In the case of testing products with antimicrobial effects, the membrane should be washed with chosen sterile solvent not less than 3 times, not exceeding the fivefold cycle of filter washing for 100 mL of solvent. The entire membrane is transferred to a suitable medium or is aseptically cut into two identical parts, which are transferred into two different media. In the case of solids soluble in water, the substance should be dissolved in a suitable solvent and the further procedure should be the same as with water solutions. The indirect method can be also used for ointments. Ointments with fatty bases can be diluted with isopropyl myristate if it is required, at the temperature not higher than 40°C. In exceptional situations, the upper temperature limit may be 44°C. Afterwards, the product is filtered as quickly as possible. For every drug form, after filtration and washing, the membrane is transferred to the medium or the medium is introduced to the filtration set on the membrane [9, 54].

#### 3.1.2. Determining pH

The pH of solutions, drops, suspensions, and* in situ* gels is most often determined using a potentiometric method. In this method, the pH value is determined by measuring potential difference between electrodes placed in examined and reference solutions of known pH or between measurement (glass) electrode and reference (calomel or silver chloride) electrode, both placed in examined preparation [9, 44, 48, 54–56].

#### 3.1.3. Clarity Examination

Clarity examination involves the visual assessment of formulation in suitable lighting on white and black background. It is performed for liquid forms, with the exception of suspensions. This examination applies to eye drops and* in situ* gels before and after gelling [54, 55].

Another method of clarity examination involves transmittance measurement using a UV-Vis spectrophotometer. This method can be employed in research on contact lenses filled with active ingredients. The lenses are hydrated in physiological saline and placed on the surface of quartz cuvette. The transmittance is measured afterwards from 200 to 1000 nm wavelength [17].

#### 3.1.4. Examination of Size and Morphology of Particles

For examination of particles' size multiple methods are employed: optical microscopy (microscopic particle count test), light obscuration particle count test, dynamic imaging analysis, laser diffraction particle analyzers, electron microscopy (SEM, TEM, AFM), DLS (dynamic light scattering), Coulter Counter test, and nanoparticle tracking analysis (NTA).

*Optical Microscopy Method (Microscopic Particle Count Test)*. Description of this method includes requirements from both American and International Pharmacopoeia. The examination is performed under microscope after taking sample, rinsing, and drying it on microporous membrane filter with pores' diameter ≤ 1 *μ*m. This examination enables calculating the number of particles sized ≥ 10 *μ*m in examined products. The test begins from small magnification, for example, ×10 or ×50, at which it is possible to find particles larger than 25 micrometers. After that, at ×100–×500 magnification, smaller particles are being searched for [9, 57, 58]. In order to fulfill requirements of American Pharmacopoeia for formulations, no more than 50 particles per mL may be of size ≥ 10 *μ*m, no more than 5 particles per mL should be of size ≥ 25 *μ*m, and no more than 2 particles per mL may be of size ≥ 50 *μ*m [59]. On the other hand, the requirements of International Pharmacopoeia stipulate that, for every 10 *μ*m of solid active ingredient, no more than 20 particles should be of maximum size larger than 25 *μ*m and no more than 2 of these particles should be of maximum size larger than 50 *μ*m. None of these particles can be of maximum size larger than 90 *μ*m. This method cannot be employed for particles' analysis in difficult to filtrate solutions of high viscosity [9].

*Light Obscuration Particle Count Test*. The examination is performed using a device which counts particles contained in liquid and employs a light obscuration sensor with a suitable system dosing the sample to provide controlled portions of sample for analysis. The suspended particles in liquid sample, floating between light source and the sensor, cause changes in signal, which are correlated with size of the particles. The nature of the system which detects and counts particles causes air bubbles, as well as drops of immiscible liquids, to block sufficient amount of light, because of which they may be recorded together with suspended particles. The influence of these factors on the measurement should be neutralised by its suitable technique. This method has certain limitations for formulations that do not exhibit lucidity and viscosity close to water. Moreover, colour formulations, as well as these with high viscosity, exhibiting changes from shear stress or forming air or gas bubbles in the moment of contact with the sensor, for example, products containing bicarbonate buffer, also generate wrong results. For such formulations, in order to measure size of particles, the membrane microscopy method is used. The equipment used for examinations of chosen formulation should have the maximum range of detected concentration (maximum number of particles per mL) larger than predicted concentration of examined formulation, whereas the dynamic range of equipment, that is, range of sizes of particles for which the size and amount may be precisely specified, must include the smallest size of particle which may be found in examined formulation [59].

*Dynamic Imaging Analysis*. This examination enables measurement of size and shape of particles in solutions or suspensions. It involves recording the digital images of particles suspended in moving fluid, for example, during mixing or flow, which enables marking the number of particles in specified volume and specifying particle size distribution. The lower range of size of particles detected by optical microscope used in dynamic imaging is about 1 *μ*m. The full particle size range possible to observe using this method is from about 1 *μ*m to over 1000 *μ*m. However, a single measurement does not enable observing particles of sizes within the whole range. While observing particles of size of lower range limit, it is not possible to observe particles of size of upper range limit. Flow-based systems differ from one another in, among others, the sampling method, the quality of digital image, percentage of simultaneously analysed particles, and the range of particles' concentration at which measurement is possible. The main advantages of digital imaging method are a real-time measurement and its conditions, in which particles remain suspended in the liquid. It allows imaging of very irregular shapes of particles and observing dynamic behaviour of particles under conditions of changing size distribution [57].

*Laser Diffraction Particle Analyzers*. The examination involves passing a laser beam through a sample containing particles of different shapes, which scatter the light, and the direction and intensity of scattered light are closely related to the size of particles in examined sample. The diffraction of light can be described mathematically using the Fraunhofer or Mie theory. The standard laser light diffraction analyzers employ detectors whose particle size measurement range is from 0.5 to 2000 *μ*m. Using a suitable technology (PIDS—polarization intensity differential scattering) enables reduction of the lower range limit of measuring instrument to even about 17 nm. One of the biggest disadvantages of laser beam scattering technology is the large sample volume. However, the size of sample is largely related to the concentration of particles—as it grows, the required sample volume falls. Analysis of sample with the use of laser diffraction analyzers often requires large dilution of samples. It is also important to point out that in most of scattered light measurements, the size of particle of examined sample is determined by calculating the equivalent spherical diameter, regardless of actual particle shape [57].

*Electron Microscopy (SEM, TEM, AFM)*. Advanced microscopic methods, such as transmission electron microscopy (TEM), scanning electron microscopy (SEM), and atomic force microscopy (AFM), enable high-quality imaging of particles in nanometer resolution. TEM and SEM require, however, strong samples processing. On the other hand, AFM enables capturing the topology of particles' surface on the image in nanometer resolution. All three methods are appropriate largely for specialistic application because of high equipment costs, low efficiency, and changing conditions of sample examination [57, 60–65].

*DLS (Dynamic Light Scattering) or Photon Correlation Spectroscopy, Quasielastic Light Scattering*. The DLS method measures fluctuations of scattered light caused by Brownian motion of molecules in a solution and is therefore related to diffusion coefficient. From the Stokes-Einstein equation, knowing the value of diffusion coefficient, it is possible to determine the hydrodynamic particle radius in examined sample. It is the radius of the sphere having the same diffusion coefficient as the measured particle. DLS enables simple and quick measurements of particles' size in the range from <1 nm to even 10 *μ*m for one marking. The examinations can be performed on solutions or suspensions of active ingredients without the need of modification or dilution of formulation for very small sample volume (10–100 *μ*L). DLS is the only method which enables measuring of particles' size in the solution in wide range, that is, from about 0.3 to over 1000 nm, which partly fills the gap of submicron analysis. This method is mostly used in batch mode with nonfractionated samples placed in cuvettes or well plates. Its disadvantages are limitations in size resolution, lack of shape measurement possibility, and masking light scattering intensity by small particles in the presence of considerable amount of larger ones. DLS can resolve two different size groups only when their hydrodynamic diameters differ 2–5 times [13, 57, 62–66].

*Coulter Counter*. This method employs the rule which says that particles placed in electric field modify the flow of charge (current). For detecting particles, the electrical sensing zone technique is used. The method of determining size and amount of particles using Coulter Counter is described in ISO 13319 norm (“Determination of particle size distribution—electrical sensing zone methods”). The particles' size measuring range in this method is from about 0.4 *μ*m to 1600 *μ*m. Several markings enable detecting particles in the whole measuring range. The main advantage of this method is the fact that particles' properties, that is, colour, shape, composition, or refractive index, do not affect the measurement. Using Coulter Counter, it is possible to obtain very precise particles' size distribution. Before the measurement, a formulation containing particles must be suspended in electrolyte, which may cause changes in composition or number of particles [57, 67, 68].

*Nanoparticle Tracking Analysis (NTA).* NTA is a new technique employed for measuring size of particles in the range from about 30 to 1000 nm. It combines laser light scattering microscopy with a CCD camera, which enables visualization and recording particles in a solution. The examination, as in the DLS method, involves determining size of the particle from Stokes-Einstein equation. NTA exhibits more precision in size distribution in comparison to DLS but requires larger volume of the sample (about 300 *μ*L) [57, 69].

#### 3.1.5. Examination of Content of Substance or Preservative

The examination of drug or preservative content in given formulation is labeled with relevant analytical technique, that is, spectrophotometric method or HPLC [12, 21, 26, 54, 55, 61, 70].

#### 3.1.6. Examination of Drug and Carrier Interaction/Compatibility Using FTIR, DSC, and XRD Methods

Fourier transform infrared spectroscopy (FTIR) and examinations employing differential scanning calorimetry (DSC) and X-ray diffractometry (XRD) are performed for, among others, pure substance, physical mixtures of drug and polymers used to obtain formulation, and the ingredients of the formulation in order to identify potential interactions between the active ingredient and other ingredients of the preparation [21, 55, 62].

#### 3.1.7. Stability Examination

The purpose of stability examination is to provide information on changes in quality of active ingredient or medicinal product in time due to the effect of environmental factors, that is, temperature, humidity, and light, on examined substance/product, as well as to set the date of further examination of medicinal substance or expiry date of medicinal product and recommended storage conditions [6].

General stability requirements for ophthalmic products, for example, drops and ointments, are similar to those for other pharmaceutical products. They are harmonized through ICH (International Conference on Harmonisation) process in USA, Europe, and Japan, acknowledging the contribution of a European institution EMEA (European Agency for the Evaluation of Medicinal Products) and its Committee for Proprietary Medicinal Products (CPMP), QWP (Quality Working Party), and the American institution FDA (Food and Drug Administration) as well as the Japanese Ministry of Health [71]. Generally, active ingredients should be stored in conditions that enable assessment of their thermal stability and, if applicable, also proneness to humidity. Storage conditions and examination period should correlate with warehousing, transport, and later use conditions [6].

There are many documents containing guidelines on stability examinations. However, they are general and often do not acknowledge special features of ophthalmic products. Matthews and Wall, in their article, referenced (with a short description) the documents which may constitute footholds for planning stability examinations of ophthalmic products, particularly those different from conventional drops and ointments [71]. General conditions for stability examination are contained in Tables 1, 2, and 3.

Despite existing guidelines, the scientists often choose their own conditions for stability examinations. Nagargoje with associates performed stability examinations for an* in situ* gel containing fluconazole at temperatures 4°C ± 1°C, 27°C ± 1°C, 45°C ± 1°C for one month period [55], and Nanjwade with associates examined eye drops stability at temperatures 5°C, 25°C, 37°C, and 45°C [54].

#### 3.1.8. Drug Release Studies

In literature, several methods employed for the examination of accessibility of pharmaceutical substance from ophthalmic forms were described. They include bottle method, modified rotating basket method, diffusion method with the use of Franz cell, modified rotating paddle apparatus, or method with the use of flow-through device.

*Bottle Method*. In this method, the examined drug forms are placed in culture bottles [72, 73] or vials [21, 38, 74, 75] containing phosphate buffer at pH 7.4 [21, 38, 72–74, 76, 77] or artificial isotonic tear fluid [75]. Bottles and vials are usually shaken in water baths [21, 38, 72–74] (or incubated under magnetic stirring [76, 77]), mostly at a temperature of 37°C [72–74, 76, 77], and the medium samples are taken in specified time intervals and examined for drug amount using a suitable analytical method [21, 38, 72–77].

*Diffusion Method with the Use of Franz Cell or Other Two-Compartment Systems*. This method employs a two-chamber system consisting of two compartments: donor and receiver. A sample of examined formulation is placed in a donor compartment of Franz cell or other systems, while a receiver compartment contains a thermostated dissolution medium, for example, at the temperature of 37°C ± 0.5°C, subjected to continuous stirring using a magnetic stirrer, usually at the speed of 50 rpm. Both compartments are separated with a dialysis membrane, for example, made from cellophane. During examination, in specified time intervals, samples of dissolution medium are taken, and the medicinal substance is marked using a suitable analytical technique [55, 56, 65, 72].

For release tests in the described method, a glass container, for example, of a cylindrical shape, may be used. It is placed in a beaker (a receiver compartment) [44, 61, 78, 79], filled with an artificial tear fluid [78, 79] or phosphate buffer at the pH of 7.4 [44, 61]. In the cylindrical container, constituting a donor compartment, an examined drug form is placed, after which a diffusion cell membrane is put on a containers aperture. The ingredients of the compartment are continuously stirred at fixed temperature using a magnetic stirrer. In specified time intervals, samples of dissolution medium are taken, and the medicinal substance is marked using a suitable analytical technique. The taken sample amount is replaced with analogical amount of a fresh solution simulating a tear fluid or phosphate buffer [44, 61, 78, 79].

*Modified Rotating Basket Method*. In this method, a drug form is placed in a set of baskets or substitutes, for example, glass cylindrical pipes, connected with a stirrer. The glass pipes are covered with dialysis membrane on one side, while the other side is attached to shafts of the apparatus. All the components are put in a beaker with a water jacket, containing a buffer solution, for example, a simulated tear fluid (STF). Temperature of the system may be maintained, for example, at 35°C ± 1°C, and the frequency of stirrer rotation may be at, for example, 50 rpm. A sample of solution is taken in specified time intervals and examined for drug amount. The taken sample amount is supplemented with the analogical amount of a fresh solution simulating a tear fluid in order to keep constant volume [80].

*Modified Rotating Paddle Apparatus*. In this method, diffusion chambers, used for analysis of half-solid formulations, are placed in a paddle apparatus container. A suitable liquid is poured into the container and stirred during test at the speed, for example, of 50 rpm, at the temperature of 37° C. Containers with diffusion chambers soaked in dissolution medium are placed in a water bath, maintaining the temperature at 37 ± 0.5°C. Samples of buffer solution into which the substance from diffusion chambers is being released are taken in specified time intervals and examined for drug content [72, 81].

Kao and associates employed this method for examination of substance release from nanoparticles introduced directly to a paddle apparatus container holding a solution simulating tear fluid, stirred at the speed of 75 rpm at the temperature of 37°C. In specified time intervals, they took solution samples, centrifuged them, and marked spectrophotometrically in a supernatant the amount of active ingredient [62].

*Flow-Through Devices.* In this technique, an apparatus in which permanent dissolution medium circulation takes place is employed for substance release studies. The device consists of a cell in which the substance is dissolved (a jacketed flow-through cell), a continuous duty oscillating pump, a water bath, and a jacketed flask containing a dissolution medium. A drug form is put in the jacketed flow-through cell, into which a dissolution medium is introduced afterwards. The medium circulates in closed cycle. Temperature is maintained at a level close to that of human body (e.g., 33 ± 2°C or 37°C) and the samples are taken in specified time intervals and are examined for drug content [72, 82].

Examinations of active ingredients release from drug forms may be performed also in flow-through devices with open flow, which was described in articles written by Rao and Shyale [83] as well as Tanwar and associates [84].

Other examinations performed for ophthalmic drug forms include viscosity examinations using viscometers [44, 48, 55, 56], osmolarity examinations using osmometers [44, 48, 56], and the light refractive index measurement using ellipsometers/refractometers [20, 48].

### 3.2. Other Examinations Performed for Chosen Drug Forms

#### 3.2.1. Examinations for* In Situ* Gels

*Examination of Gel-Forming Ability.* This examination is performed in order to assess the ability of formulation to form gels on the surface of eyeball. A sample of examined formulation is introduced to a vial containing a solution whose components simulate a tear fluid and visual technique is employed to assess the sol-gel phase transition [55, 61].

#### 3.2.2. Examinations for Inserts

*Swelling Index.* Hydrophilic polymers of different structures exhibit different swelling degree, depending on relative resistance of matrix network structure to water particles' movement. Polymer chains exhibiting low ability to form hydrogen bonds may not be able to form strong network structure, resistant to fast water penetration. The bigger the strength and number of hydrogen bonds between polymer chains are, the slower the water particles diffuse into the hydrated matrix. Swelling of the polymer is vital to activation of bioadhesive abilities, which activate just after swelling begins. With the growth of polymer hydration, the adhesion grows until the moment when excessive hydration leads to sudden fall of adhesion strength, which is an effect of the untangling of outer polymer layer. The degree and speed of insert hydration, as well as swelling, affect drug release from a dosage form. Therefore, this parameter is of greatest significance for drug release prediction and bioadhesive matrix potential. Swelling examination is performed to measure bulk hydrophilicity and polymer hydration [21]. In the procedure, a specified number of inserts are chosen, weighed, and put separately in beakers containing a solution simulating tear fluid [78], physiological saline buffered with phosphates [21], or distilled water [85] at fixed temperature, for example, 32°C ± 0.5°C [21]. In specified time intervals, inserts are taken out, dried with filter paper, and weighed once more. The procedure is repeated until the moment when mass growth is not observed anymore [21, 78, 85]. The degree to which the liquid is taken up, called the swelling index, is calculated from the formula

(1) $$\begin{array}{c} \text{Swelling index}=\left[\frac{\left(W_{t}-W_{0}\right)}{W_{0}}\right]\times 100, \end{array}$$

where *W*_0_ is the initial sample weight and *W*_*t*_ is the sample weight at *t* time [21].

*Examinations of Moisture Absorption and Loss.* These examinations are performed in order to assess physical stability and integrity of inserts' polymer matrix in dry conditions and at raised moisture [21, 85].

For moisture absorption examination, a specified number of inserts are chosen and placed in desiccator, in which high moisture level, for example, 75 ± 5% RH, is maintained. After a specified time period, inserts are taken out and weighed again, and the percentage moisture absorption is calculated from the formula [21, 70, 85]

(2) %  Moisture Absorption  =(Final weight−Initial Weight)×100Initial Weight.  

In moisture loss examination, a chosen number of inserts are put in desiccator containing anhydrous calcium chloride, which ensures dry conditions inside the container. After a suitable time period, inserts are taken out and weighed again, and the percentage moisture loss is calculated from the formula [21, 85]

(3) $$\begin{array}{c} \%\;\text{Moisture Loss}=\frac{\left(\text{Initial Weight}-\text{Final weight}\right)\times 100}{\text{Initial Weight}}. \end{array}$$

For eye inserts assessment, examinations of thickness [26, 70, 79] and weight uniformity [26, 70], as well as mechanical strength tests [70, 79], are also advisable.

#### 3.2.3. Examinations for Multicompartment Drug Delivery Systems

*Encapsulation Efficiency*. A sample for encapsulation efficiency examination is obtained by centrifuging [62, 64, 65] or centrifugal ultrafiltration [13, 44] of mixture formed after preparing the formulation. The obtained supernatant or filtrate is examined for amount of free active substance using a spectrophotometric method [44, 62, 64] or HPLC [13, 65]. Encapsulation efficiency is calculated from the formula

(4) $$\begin{array}{c} \text{E}.\text{E}.\;\left(\%\right)=\;\left(W_{\text{total}}-W_{\text{free}}\right)\times \frac{100}{W_{\text{total}}}, \end{array}$$

where *W*_total_ is the total amount of drug in the formulation; *W*_free_ is the amount of drug in the filtrate/supernatant [13, 62].

### 3.3. *In Vivo* Examinations

#### 3.3.1. Eye Irritancy Test (Draize Eye Test)

There are many modifications of eye toxicity/irritancy test (Draize eye test) performed for dosage forms, that is, solutions, emulsions, ointments, solids, for example, inserts, and so forth. Examinations are usually carried out on rabbits, whose vision organ anatomy and physiology are well described in literature. Moreover, rabbits' eyes are usually more susceptible to irritating compounds than those of humans. For the test, usually from 3 to 6 rabbits are used, which, on one hand, enables obtaining reliable results, and, on the other hand, is an answer to claims for applying toxic substances to as little animals as possible. The most often used animal subspecies are albino (e.g., New Zealand) rabbits, which are examined and weighed before the test and then placed in specifically adapted cages, designed so as to avoid accidental injuries. The examined preparations are introduced to conjunctival sac or applied directly on the cornea. At first, about 0.1 mL of analyzed drug was being applied on the eyeball, but many later examinations pointed to reducing the amount, for example, to 0.01 mL, which more reflects real situations. In the test, one eyeball, usually the left one, is used as a control. After introducing a drug form on the eyeball, the eyelids are usually kept closed for a few seconds, although it is not required. Sometimes sterile solutions are additionally used for rinsing the eyeball surface. An assessment of eyeball condition before and after introducing the formulation is done by observation of the eyeball in suitable light, often using magnifying glass or a slit lamp, which ensures more precise evaluation. Auxiliary procedures which simplify visualization of changes include dyeing with fluorescein and taking photos of eyeball. Moreover, the discomfort level after application may be indicated by the number of blinkings or rubbings of the eye. The evaluation takes place usually after 1 h, 24 h, 48 h, and 72 h from introducing a drug form on the eyeball and, if essential, also after 7 or 21 days. Duration of examination, as well as its scheme, is individually adapted to the analyzed formulation. Ocular changes are assessed using a scoring system, in which every change in the area of eyelid, conjunctiva, cornea, and iris is scored. While in literature many scoring systems were proposed, the modified Friedenwald and Draize methods are still widely employed [21, 48, 54–56, 64, 86].

#### 3.3.2. Transcorneal Permeation Study

For transcorneal permeation study, as in the Draize eye test, healthy albino rabbits are chosen in the number which is suitable for obtaining reliable results. The amount of active substance in aqueous humor after introducing the formulation to conjunctival sac is marked in specified time intervals. Using a syringe with needle, after intramuscular or intravenous anaesthetic injection which may contain, depending on application, ketamine hydrochloride, xylazine hydrochloride, or pentobarbital sodium, a sample of aqueous humor is taken in the amount of about 150–200 *μ*L and stored at negative temperature, for example, −20° C, before HPLC analysis [13, 64, 80–82]. At times, additional inhalation anaesthesia is used, for example, in the form of mixture of 4% isoflurane-oxygen, shortly before or during paracentesis [64]. Regional anaesthesia, for example, in the form of xylocaine solution, may also be applied [81]. Noomwong with associates, during performed tests, added suitable amount of 2% ZnSO_4_·7H_2_O solution to the taken samples in order to salt out proteins contained in aqueous humor and then centrifuged the sample at the speed of 10000 rpm for 1 h at the temperature of −10°C. They used HPLC method to examine the amount of active ingredient in the obtained supernatant [64]. On the other hand, El-Laithy et al. and associates examined obtained samples using a spectrofluorometric method, which could have been employed due to natural fluorescence of used drug from fluoroquinolone group, moxifloxacin [80].

#### 3.3.3. *In Vivo* Release Evaluation of Inserts

For* in vivo* release evaluation, formulations which gave desired results in* in vitro* release evaluations are chosen. Inserts are put in conjunctival sacs of healthy rabbits chosen for studies. In specified time intervals, inserts are carefully taken out and examined for left drug amount using a suitable analytic technique [24, 26, 81, 84].

## 4. Conclusions

Despite many achievements in the field of ophthalmic dosage forms, still vast majority of active substances for use in ocular disorders are in the form of eye drops. Some of the more complex forms appeared on the pharmaceutical market, such as Ocusert by Alza Corporation, but scientists are still looking for the perfect ophthalmic system, which would possess desired properties such as controlled release, minimizing systemic effects, ease of use, and extended retention time at the site of application. Multicompartment systems appear to be promising drug forms that can also be combined with other forms, for example, polymeric nanoparticles with the active substance suspended in the* in situ* gel.

In connection with the development of new ophthalmic dosage forms, a problem concerning the analysis of their physicochemical properties and* in vitro*-*in vivo* correlation appears. This paper is a review of the available literature which allows planning studies to be conducted on standard and modern ophthalmic drug forms.

## Figures and Tables

**Table 1:** General conditions for stability examination [6].

Study	Storage conditions	Minimum time period covered by data at submission
Long term*	25°C ± 2°C/60% RH^1^ ± 5% RH or 30°C ± 2°C/65% RH ± 5% RH	12 months
Intermediate**	30°C ± 2°C/65% RH ± 5% RH	6 months
Accelerated	40°C ± 2°C/75% RH ± 5% RH	6 months

^1^Relative humidity.

*It is up to the applicant to decide whether long-term stability studies are performed at 25 ± 2°C/60% RH ± 5% RH or 30°C ± 2°C/65% RH ± 5% RH.

**If 30°C ± 2°C/65% RH ± 5% RH is the long-term condition, there is no intermediate condition.

**Table 2:** Conditions for active ingredients stored in refrigerators [6].

Study	Storage conditions	Minimum time period covered by data at submission
Long term	5°C ± 3°C	12 months
Accelerated	25°C ± 2°C/60% RH ± 5% RH	6 months

**Table 3:** Conditions for active ingredients stored in freezers [6].

Study	Storage conditions	Minimum time period covered by data at submission
Long term	−20°C ± 5°C	12 months
